# The Sierra Leone Ultrasound Rainbow4Africa Project (SLURP): an observational study of ultrasound effectiveness in developing countries

**DOI:** 10.1186/s13089-016-0051-y

**Published:** 2016-10-26

**Authors:** Alessandro Lamorte, Enrico Boero, Paola Crida, Abdul R. Conteh, Marco Foletti, Paolo Narcisi

**Affiliations:** 1 Emergency Department, San Luigi Gonzaga Hospital, Regione Gonzole 10 Orbassano, 10043 Turin, Italy; 2 Department of Surgical Sciences, Turin University, Turin, Italy; 3 Turin University, Turin, Italy; 4 Holy Spirit Hospital, Makeni, Sierra Leone; 5 Rainbow for Africa NGO, Via Zuretti 29, Turin, TO Italy

**Keywords:** Point-of-care ultrasound, Low-income countries, Developing Countries, Rural context, Emergency medicine, Diagnostic, Clinical decision making, Abdominal ultrasound

## Abstract

**Background:**

Diagnostic tools available in low-income countries are often really basic even if patients can be as sick as those of the richer countries. Point-of-care ultrasound could be a solution for this problem. We studied the impact of ultrasound at the Holy Spirit Hospital, Makeni, Sierra Leone.

**Methods:**

This is a prospective, observational study on outpatients presenting at the HSH. We enrolled continually for 1 month 105 patients asked for ultrasound examination by the caring physician that had to indicate the differential diagnosis hypothesized, the confidence degree about these on a 5-point Likert scale, and the therapy before and after the US. The primary outcome was to measure the difference in the number of differential diagnoses. Secondary outcomes were the rate of new diagnoses, the confidence changes of the visiting physician, and the changes in prescribed therapy or management. Categorical variables were compared using the Chi-square test, and continuous ones using two-tailed Student’s test and Likert with the Wilcoxon rank-sum test.

**Results:**

194 differential diagnoses were formulated, with a mean of 1.85 (DS 0.87) diagnoses per patient. 89 (46%) were excluded on the basis of US, reducing the mean of differential diagnosis per patient to 1, 0 (*p* < 0.001). US also introduced 53 new diagnoses in 42 patients (mean 1.26; SD 0.54), raising the final differential diagnosis from 105 to 158 (+50.5%) that is 1.51 (DS 0.79) per patient. There is a statistically significant reduction (18.6%) in diagnoses per patient after having performed the ultrasound (*p* < 0.001). The certainty level increased (Wilcoxon rank-sum test: *p* < 0.001). We did not reach the statistical significance studying the changes in therapy and management because the subgroups for analysis were too small. Nonetheless, we saw interesting changes in drug prescription and referral rate before and after the US.

**Conclusion:**

Ultrasound is feasible in low-income countries; with it diagnostic hypotheses were reduced and new unexpected diagnoses were introduced. Further studies are needed to explore other strong outcomes like mortality, length of stay in hospital, and money saved with the use of ultrasound in developing countries.

**Electronic supplementary material:**

The online version of this article (doi:10.1186/s13089-016-0051-y) contains supplementary material, which is available to authorized users.

## Background

The struggle to diagnose and treat diseases is usually one of the hardest. But it can be worse in a low-resource country where Diagnostic tools available are really basic especially for doctors used to the western standards of technology. Especially if we consider that the low-income countries patients can be as sick as those of the richer countries.

Sierra Leone has been dramatically influenced by a decade of civil war that affected over two million people. The country is among the last countries in the Human Development Index and has a mortality rate of 36.8% in adults. The main pathologies present in the country are malaria, tuberculosis, and HIV. Access to health services is difficult and very often patients must pay for these services and travel for long distances on foot, or face new charges for transport. So much of the population turns to traditional healers, which in many areas of the country are the only chance of cure.

Ultrasonography and specifically point-of-care ultrasound (POCUS) could be a solution for this lack of diagnostic possibilities because of some intrinsic features of the technology. Ultrasonography (US) is cheap, dynamic, repeatable, bedside (if the machine can be moved or has a battery), and can answer any questions the physician could have. To date, ultrasonography was used almost in every condition, from war to the Amazon jungle to Tibet [1, 2], from space to Everest [3, 4], and has shown to fit in all of these conditions.

We planned to study the impact of the ultrasound performed by a specifically trained nurse at the Holy Spirit Hospital, Makeni, Sierra Leone. Our primary outcome was to measure the difference in the number of differential diagnosis before and after US. Secondary outcomes were the rate of introduction of new unexpected diagnoses after the US exam; the confidence changes of the visiting physician in diagnoses hypothesized before and after the US; and the last point was to evaluate changes in prescribed therapy or inpatient management (surgical referral, medical only therapy).

## Methods

### Study design and sample selection

This is a prospective, observational study on outpatients presenting at the Holy Spirit Hospital of Makeni in Bombali district, Sierra Leone. Patients were continuously enrolled from April 28, 2014 to May 21, 2014 from the outpatient department (OPD) where four physicians examined them and indicated the need for an ultrasound exam. All patients requiring an ultrasound examination, as directed by the physician on duty, were eligible for enrollment. Exclusion criteria were age <18, refusal to participate by the patient. Each enrolled patient was given an explanation of the nature and purpose of the study and a written information sheet before collecting the consent (Additional file 1). The ultrasound examination was always performed by a local skilled sonographer (ARC) under the supervision of a Rainbow 4 Africa sonographer (AL, EB) experienced in lung, abdomen, and heart ultrasound. All ultrasound examinations were carried out with a Toshiba Memio 20.

### Study protocol and data collection

Common demographic variables were collected. The visiting physician was asked to fill out the first page of the case report form (CRF, Additional file 2) indicating the main complaint(s) of the patient, the differential diagnoses hypothesized (choosing from a list of the most typical diagnoses based on data from the previous year of US exam by ARC), the confidence degree about these diagnoses on a 5-point Likert scale and which area of therapy would have fit the patient’s problems (i.e., medical, surgical, or the need for a referral). The clinician was able to ask for several US scans: cardiac, chest (i.e., pulmonary), abdominal, ob-gyn, and soft tissue, or a combination of them, according to proposed differential diagnosis; this selection was recorded too. The US exam was then performed in the room next to the examination one by a nurse who was the only staff trained in point-of-care ultrasound available. The sonographer was aware of the main complaints and formulated differential diagnoses. He reported the findings on the standard form used in the hospital to give feedback to the caring physician. After receiving the report, the visiting physician was asked to fill in the second page of the CRF, indicating the new list of differential diagnosis for the patient, if any new diagnosis had come up (yes/no), the final degree of confidence using the same 5-point Likert scale and the final therapy. The full database is available in Additional file 3.

### Statistical analysis

Descriptive analysis was carried out using common measures of synthesis; values are expressed as mean ± standard deviation (SD). Categorical variables were compared using the Chi-square test; Likert scales were compared with the Wilcoxon rank-sum test. Continuous variables were compared using two-tailed Student’s t test after determining homoscedasticity. Statistical significance was set at 5%.

## Results

**Fig. 1:**
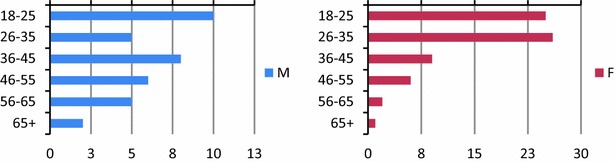
Relative frequencies of age classes among male (*left panel*) and female (*right panel*) population

From April 24 to May 24, 2014, 105 patients were enrolled from the OPD of the Holy Spirit Hospital. Among them, 69 patients were female (65.7%) and the majority of the enrolled patients (62.9%) were less than 35 years old (Table 1; Fig. 1). 105 ultrasound exams were requested by four different physicians, asking, respectively, for 62, 21, 20, and 2 exams. The physician who requested the higher number of exams was also the one who visited the majority of the patients.

**Table 1:** Overall relative frequencies of age classes among entire population and prevalence of genders

	N (%)
Sex	
Male	36 (34.29)
Female	69 (65.71)
Overall	105 (100)
Age	
18–25	35 (33.33)
26–35	31 (29.52)
36–45	17 (16.19)
46–55	12 (11.43)
56–65	7 (6.67)
66+	3 (2.86)
Overall	105 (100)

### Ultrasound exam requests

The most requested ultrasound exam was the abdominal one (43), followed by transabdominal obstetric-gynecological (35), combined chest and abdominal (22), soft tissue (4), and cardiac exam (1). The stratification of the requested exam according to the treating physician’s level of confidence prior to the exam itself highlighted how ultrasound scans limited to the abdomen only were requested when facing higher level of confidence, while combined chest and abdominal scans where requested in case of lower levels of confidence (χ^2^ = 139.9; 16 g.l.; p < 0.001; Table 2).

**Table 2:** Type of ultrasound exam requested according to the relative level of confidence of the attending physician

Type of ultrasound exam	Level of confidence before US					Totals
	1	2	3	4	5	N (%)
Cardiac	1	0	0	0	0	1 (0.9)
Chest–abdomen	0	12	9	0	1	22 (20.9)
Abdomen	0	7	24	11	1	43 (40.9)
Ob/gyn	0	10	5	19	1	35 (33.3)
Soft tissues	0	3	1	0	0	4 (3.8)
Totals	1	32	39	30	3	105 (100)

### Main complaints and differential diagnosis

The most frequent symptom was abdominal pain (60 cases, 63%) followed by obstetric-gynecological problems (i.e., menstrual irregularities, vaginal discharge, other obstetric problems: 21 cases, 20%). Most of the patients (78%) reported more than one complaint. Table 3 shows all the complaints that led the patients to the hospital.

**Table 3:** Total amount of presenting symptoms in the sample and their distribution among male and female

Symptoms	Overall N (%)	Male	Female
Chest pain and burns	22 (12)	13	9
Cough	5 (3)	5	0
Dyspnea	7 (4)	6	1
Palpitation	5 (3)	2	3
Swelling of feet	8 (4)	4	4
Abdominal pain	64 (34)	23	41
Abdominal distension and discomfort	11 (6)	6	5
Feeling of abdominal mass	10 (5)	5	5
Swollen testes	1 (1)	1	0
Difficulty in passing urine	3 (2)	1	2
Obstetrics problems	16 (9)	0	16
Menstrual problems	20 (11)	0	20
Vaginal discharge	15 (8)	0	15
Total	187 (100)	66	121

Considering all the patients, the treating physicians *hypothesized* an amount of 24 possible pathologies in formulating the differential diagnoses before the US exam. The most frequent was genitourinary tract infection or adnexitis (34 cases), followed by peptic ulcer disease (27) and pregnancy (17). Such diagnoses remained the most represented even after the US examinations, though with a different frequency (Table 4).

**Table 4:** Suspected diagnosis in the sample

Suspected diagnosis	Before-US N (%)		After-US N (%)		Ruled out N (%^a^)		Added N (%^b^)	
Pneumonia/TB	9	(4.6)	3	(1.9)	8	(88.9)	2	(66.7)
Pleural effusion	5	(2.6)	4	(2.5)	3	(60.0)	2	(50.0)
Pulmonary overload	0	(0.0)	3	(1.9)	0	–	3	(100)
Cardiomyopathy	12	(6.2)	6	(3.8)	7	(58.3)	1	(16.7)
Aortic ectasia	0	(0.0)	2	(1.3)	0	–	2	(100)
Liver cirrhosis/schistosomiasis	12	(6.2)	12	(7.6)	8	(66.7)	8	(66.7)
Cholecystolithiasis	0	(0.0)	1	(0.6)	0	–	1	(100)
PUD	27	(13.9)	24	(15.2)	6	(22.2)	3	(12.5)
Gastritis/gerd	10	(5.2)	7	(4.4)	5	(50.0)	2	(28.6)
Gastroenteritis	6	(3.1)	5	(3.2)	2	(33.3)	1	(20.0)
Appendicitis	2	(1.0)	0	(0.0)	2	(100.0)	0	–
Acute/chronic nephritis	7	(3.6)	5	(3.2)	3	(42.9)	1	(20.0)
Nephrolithiasis/hydronephrosis	2	(1.0)	1	(0.6)	2	(100)	1	(100)
Abscess/mass	11	(5.7)	16	(10.1)	4	(36.4)	9	(56.3)
BPM	0	(0.0)	1	(0.6)	0	–	1	(100)
Pelvic muscle syndrome	5	(2.6)	3	(1.9)	2	(40.0)	0	(0.0)
Splenomegaly	5	(2.6)	4	(2.5)	4	(80.0)	3	(75.0)
Musculoskeletal pain	2	(1.0)	4	(2.5)	1	(50.0)	3	(75.0)
Gynaecomastia	0	(0.0)	1	(0.6)	0	–	1	(100)
Adnexitis/gut infection	34	(17.5)	25	(15.8)	11	(32.4)	2	(8.0)
Ovarian cyst	16	(8.2)	8	(5.1)	11	(68.8)	3	(37.5)
Uterus myomatosis	7	(3.6)	2	(1.3)	6	(85.7)	1	(50.0)
Miscarriage	5	(2.6)	3	(1.9)	2	(40.0)	0	(0.0)
Pregnancy	17	(8.8)	18	(11.4)	2	(11.8)	3	(16.7)
Total (%)	194	(100)	158	(100)	89	(45.9)	53	33.5

First column: before ultrasound, based on clinical presentation. Second column: after the requested ultrasound scan and considering both the clinical and sonographical data. Third column: number of initial hypothesis excluded due to the ultrasound findings. Fourth column: number of final hypothesis added due to the ultrasound findings

^a^Percentage of ddx pre-US that has been excluded on US basis

^b^Percentage of ddx post-US that has been added on US basis

In 41 cases, the number of differential diagnoses reduced after the US, in 51 it remained the same (being 34 of them cases in which the caring doctor hypothesized only one diagnosis), and only in 13 cases the number of differential diagnosis increased after the US (Table 5).

**Table 5:** Variation of number of hypothesized differential diagnosis showed by coupling the figure before and after ultrasound scan (in the first coloumn the number of diagnosis before the US)

	0	1	2	3	4	Total
1	2	34	6	–	–	42
2	–	22	15	3	3	43
3	–	4	8	2	1	15
4	–	1	3	–	–	4
5	–	–	1	–	–	1
Total	2	61	33	5	4	105

For the whole 105 patients, a total amount of 194 differential diagnoses were formulated, with a mean of 1.85 (DS 0.87) diagnoses per patient at the first medical contact, with a statistically significant reduction to 1.51 (DS 0.79) diagnoses per patient after having performed the ultrasound (*p* < 0.001). The total amount of differential diagnoses after the US was 158, showing a reduction of 18.6%. This is a gross computation that can be divided in more subtle phenomena.

1. Out of the total amount of 194 hypotheses, 89 (46%) were *excluded* on the basis of US, reducing the mean of clinically based differential diagnosis per patient to 1.0 (*p* < 0.001). On the other hand, US *introduced* 53 new diagnosis in 42 patients (mean 1.26; SD 0.54), raising the final figure of total differential diagnosis from 105 to 158 (+50.5%).
2. In 51 cases, the number of differential diagnosis* remained the same*, due to two different mechanisms. 32 of these patients had only 1 or 2 clinical hypothesized differential diagnosis (mean 1.22; SD 0.42), and neither rule-out nor introduction of new diagnosis was due to ultrasonography. The other 19 patients had a mean of 1, 15 new differential diagnosis introduced by ultrasonography and the same amount of rule-outs. This second group of 19 patients presented to US exam with an amount of differential diagnosis before ultrasound that was significantly higher in comparison to the other 32 (p = 0.01).
3. 13 patients had their amount of differential diagnosis *increased* and it was always due to the introduction of new hypotheses by ultrasonography, with a mean of 1.6 for each patient; 5 of them also had 1 rule-out.
4. The amount of differential diagnosis *decreased* in 41 patients; they had an average of 1.51 rule-outs and 10 of them had a new hypothesis introduced by ultrasonography.

In summary, we found that 46% of 194 initial differential diagnoses were ruled out by US and that 33% of the final differential diagnoses considered were given by US. These percentages were calculated for each pathology (Table 4), showing that some pathologies had a high rate of exclusion (e.g., pneumonia/TB, splenomegaly, uterine myomatosis, ovarian cyst, cirrhosis, gastritis/PUD). Some other possible diagnoses were completely ruled out (i.e., appendicitis and nephrolithiasis). As mentioned, some diagnoses were added only after the US, like abdominal aortic dilation, pelvic mass, cholecystolithiasis, gynecomastia e pulmonary overload.

Finally, the number of differential diagnoses was not linked with the sex (*χ* ^*2*^ = 6.77; 4 df; *p* = 0.15), or with the age of the patients (*χ* ^*2*^ = 21.35; 20 df; *p* = 0.37).

New diagnoses were found in 42 patients, of whom 20 in male and 22 in female, even if our sample was composed by two-thirds of females. This means that the new diagnosis rate in males was two times higher than in female patients.

The thoracic-abdominal ultrasound gave the caring physician proportionally more new diagnoses than the abdominal and the cardiac alone (χ^2^ = 9,69; 3 df; p = 0.046).

### Physician level of confidence

**Fig. 2:**
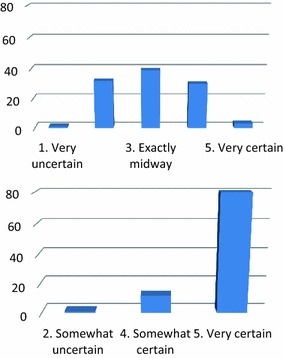
Distribution of level of confidence on the formulated differential diagnosis before the result of the ultrasound scan and after

The confidence level of the caring physicians before the US exam was distributed symmetrically around the modal value of 3. After the US, the distribution changed with a modal value of 5 asymmetrically distributed. The certainty level always increased (Wilcoxon rank-sum test: *p* < 0.001) except in 5 cases, in which it remained the same (Fig. 2).

### Therapy

The therapy proposed in every patient was recorded before and after the US, as described in Table 6.

**Table 6:** Variation of the class of therapy (medical, surgical, or both) after the ultrasound results and variation of hypothesis

Kind of therapy (pre-US)	Medical (post-US)	Med and surg (post-US)	Referral (post-US)	Surgical (post-US)	Total (pre-US)
Medical	74	3	3	2	82
Medical and surgical	3	0	0	1	4
Surgical	11	4	0	4	19
Total (post-US)	88	7	3	7	105

We analyzed both the main area of therapy (i.e., medical, surgical, or referral to other hospital) and the drugs that were prescribed for each case. After the US exam among the 82 patients who were hypothesized to be treated only medically, 3 were referred (3, 6%) and 5 were treated also or only with surgery (6%). Conversely, surgical therapy remained the only one in 4 out of the 19 patients who would have had it (21%); 11 had a medical therapy and 4 a combined medical-surgical therapy. Finally, all of the 4 patients who were thought to need a combined therapy were addressed to a medical alone or a surgical therapy alone. Getting deep into the drug prescription changes after the US exam and clinical re-evaluation, we highlighted substantial differences in prescription for some classes of drugs.

- Antibiotics: we recorded a steep increase (+182%) in antibiotic prescription and antiparasitic drugs with the exception of metronidazole (−10%) and clarithromycin (−8%) in the eradicant therapy for H. pylori-related gastritis.
- PPI: we observed a 20% reduction in omeprazole prescription, according to a reduction in PUD or Gastro-Esophageal Reflux Disease (GERD) diagnosis of 17 out of the initial 37 (rule-out 45%), and then 11 (35% of final figure) were added after US.
- NSAIDs: we observed a 133% increase in NSAID prescription.
- Congestive heart failure drugs: we observed a moderate reduction (−21%) in furosemide and digoxin prescription related to the reduction in cardiomyopathy diagnosis made using US.

## Discussion

The construction of this study is based on ample literature that indicates how ultrasound can be considered the imaging technique of choice both in the ordinary European hospital environment and, thanks to its low cost and easy transportability, in low-resource contexts such as intra/extra-hospital emergencies or healthcare in developing countries. Another important reason is the consideration that the relationship between medicine and the population of developing countries is often influenced by cultural backgrounds rooted in distant traditions that clash with Western medicine’s invasiveness. Ultrasound can therefore fill the role of a modern magic mirror that allows the doctor to “look inside” the patient without breaking through the “protective barrier” that surrounds the patient according to the animist culture. For this reason, it is often well accepted by people allowing for a great compliance between doctor and patient. Moreover, portability, costs, and steep learning curve on how to use the ultrasound machine make ultrasonography the most feasible imaging technique in this context.

As far as concern our results, abdominal pain was the most frequent symptom of presentation. In Sierra Leone, the only CT is a 3-hour drive from the HSH and the only imaging test locally available is plain X-ray, that is far less sensitive in the abdominal pain diagnostic pathway [5]. Taking this into account, US drastically improved the diagnostic possibility in our context. Moreover, the strongest barrier against the feasibility of US is not a big deal in Sierra Leone with only 3% of the population with a BMI >30 [6].

As mentioned in the results, most of the patients (78%) reported more than one complaint. In this situation, it is very likely to have a broad spectrum of differential diagnosis in which the US has been of paramount importance in leading to their reduction. In our primary outcome, it seems that with the US exam we obtained only a slight reduction in differential diagnoses from 1.8 diagnoses per patient to 1.5. Actually, we had a rule-out rate of 45% of pre-US diagnoses with 33% of new post-US diagnoses. This high turnover has led to the final result that can therefore be considered impressive. Moreover, the introduction of new potential and clinically unexpected diagnoses can be a valuable point since it can changes the management of the patient.

We noticed that the half of new unexpected diagnosis came in male patients, but male were only one-third of our sample. For this, the percentage of new unexpected diagnosis among men is nearly double than among women (56% for male, 32% for women, *χ* ^*2*^ = 5.5; 1 df; *p* < 0.02). This could be quite surprising if we consider that one would expect to find with the US something unexpected in a female abdomen due to the greater amount of organs and therefore make the differential diagnosis of abdominal symptoms harder. Something was missing also in the possible explanation, given that abdominal presentation was fourfold in females rather than males. Analyzing our sample, we tried to find the confounding variable dividing abdominal or non-abdominal patients on the basis of the presenting symptom. Observing the odds ratio of new diagnosis, it came out that abdominal presentation did not conduce to greater number of diagnosis as being male did instead (OR 3.2; CI 1.04.9.81). This tells us that male presentation was probably a risk factor itself. The explanation could lay in two possible facts that we are not completely able to analyze: male patients came with worse clinical pictures in comparison with women or the visiting doctor formulated less diagnostic hypotheses when facing a man. This second explanation came out to be unlikely, since the average of differential diagnosis was not related to sex and average of new unexpected diagnosis was not correlated to the number of differential diagnosis.

The non-homogeneous distribution of new differential diagnosis prompted by the US thoraco-abdominal exam in comparison to sole abdominal or sole cardiac has to be related with the request of the complete abdominal-thoracic exam in the cases in which the physician was less sure of his diagnostic hypotheses.

Another strong point of US is the possibility to make some final diagnoses which would otherwise be impossible. For example, in two cases we have diagnosed an aortic dilation that has led to a follow-up for the patient. In the same way, we have diagnosed one case of nephrolithiasis (rare in African population) and one of cholecystolithiasis which could not have been recognized without the US.

Analyzing the differences in therapy before and after the US examination, we can underline some issues. As far as the therapeutic strategy is concerned, we can notice how 74% of unnecessary surgeries expected before the US have been avoided. On one hand, without US three cases would not have been referred in the right hospital and two patients would not have had the early surgery they needed. On the other hand, the changes in drugs prescription, the decrease in PPI, and eradication therapy can be explained with the PUD and gastritis diagnoses reduction. These diagnoses were typically general and not specific, made by the caring physician when there were no clear features of the abdominal pain referred by the patient. With the US exam, we increased the diagnostic performance of the caring physician on the abdominal pain, the most frequent main complaint. The most important variation between pre- and post-US therapy we observed was in NSAID prescription and was probably due to the after-US new diagnoses of painful abdominal masses, abscesses, and muscular pain. In the end, although the diagnosis of cardiomyopathy decreased after the US exam, the increase in spironolactone prescription can be explained with the increase in cirrhosis diagnosis.

This study presents some merits and limitations. Its one-hospital setting cannot be clearly representative of the whole population of all low-income countries; moreover, even in low-income countries there are differences in distribution of healthcare facilities and Sierra Leone itself represents one of most deprived countries. Despite an intuitive and consolidated idea of ultrasound utility in both high and low-income countries, the ability of the physician to give the right indication for US remains a big problem to deal with. Our hospital may have had physicians that are probably different in number, experience, and ability from the physicians of another service; specifically our visiting doctor were one young general practitioner from Pakistan, one older general practitioner from Nigeria, one surgeon, and one pediatrician. One big limit is due to the absence of a certain diagnosis to compare the variation in differential diagnosis to a standard reference. This problem is widespread and, in a certain sense, it will persist in those contexts in which diagnostic facilities, whether ultrasound or other, are unaffordable to people and services. Another limit is that, even with a good sample size, our 105 patients are not enough to make reliable deductions on the single pathologies encountered, since each pathology accounts for very few cases. We were not able to fulfill the typical features of point-of-care ultrasound [7] given that our examinations were not made directly by the caring physician. This was due to the lack of doctors trained in ultrasound at all. Actually, we decided to train a nurse in ultrasound for the low doctor/population ratio of Sierra Leone that amounted at 2.2 every 100,000 population [8]. For this reason, many medical competencies, that in Western countries are doctors’ prerogative, in Sierra Leone can often be guaranteed by nurses, especially in rural zones. Finally, since US examination can be reassuring, the level of confidence of the visiting physician may have also risen because of this.

On the other hand, this study provide an example of well-integrated clinical and imaging diagnostic service, in which a point-of-care level of ultrasound facility was implemented by the collaboration between the visiting physician on duty and the sonographer, matching their ideas on the same patient in nearly real time. Both the reduction and emergence of new unexpected diagnoses are important parts of the diagnostic process and US showed to be a useful means to face this step. The confidence level of the caring physician is a point of great importance, since physician–patient relationship cannot relinquish a certain grade of confidence on the most probable diagnosis. In this context also blood tests are very expensive and limited in spectrum. Since US could aid to complete diagnostic needs, the introduction of widespread US could improve the diagnostic accuracy of local physician. The possibility to indicate an appropriate treatment instead of another, in particular a surgical one, is of paramount importance, from the point of view of safety, prognosis, and economic affordability. Finally, to our knowledge this is the first study exploring the gain in diagnostic accuracy after an ultrasound examination made by a specifically trained local nurse practitioner. In contexts with a severe lack of physician, like Sierra Leone, the results of our research highlight the usefulness of US training programs, which can be extended to all low-income countries.

Further studies are needed to explore other strong outcomes like mortality, length of stay in hospital, and money saved with the implementation of ultrasound services in low-income countries. Moreover, the Ebola epidemic brought many humanitarian resources to Sierra Leone, which could be useful to arrange a multicentric study, similar to this one, that can strengthen the results of this study.

## Conclusions

Ultrasound is feasible and practical because it is economic, repeatable, feasible, safe, portable, non-invasive, and actually well tolerated from a local anthropological point of view. It can work with electricity generators, using batteries and in difficult fields.

With ultrasound in a low-income country with a very low level of diagnostic and imaging facilities, clinical-based diagnostic hypotheses were strongly reduced and new unexpected imaging-based diagnostic hypotheses were introduced.

With the use of US, the level of confidence of the visiting physician on the final diagnosis of difficult patients rose.

The indication for a surgical treatment was reduced and global appropriateness augmented.

## Additional files

**Additional file 1.** Informed consent form.

**Additional file 2.** HSH ultrasound data sheet.

**Additional file 3.** Full database.

## Abbreviations

HSH: Holy Spirit Hospital
HIV: human immunodeficiency virus
POCUS: point-of-care ultrasound
US: ultrasound
OPD: outpatient department
CRF: case report form
SD: standard deviation
PPI: proton pump inhibitor
PUD: peptic ulcer disease
GERD: gastro-esophageal reflux
NSAID: non-steroidal anti-inflammatory drugs
CT: computed tomography
BMI: body mass index
