# Supplementary material for: The Sierra Leone Ultrasound Rainbow4Africa Project (SLURP): an observational study of ultrasound effectiveness in developing countries
Source: Crit Ultrasound J. 2016 Oct 26;8:14. doi: 10.1186/s13089-016-0051-y (PMC5080275; doi:10.1186/s13089-016-0051-y)
Supplement: Supplementary file 1 — Additional file 1. Informed consent form. [file 13089_2016_51_MOESM1_ESM.docx]

***
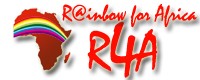
***

## INFORMED CONSENT FORM

# Sierra Leone Ultrasound Rainbow4Africa Project (SLURP)

Principal Investigator: Alessandro Lamorte MD

Co-Investigator: Abdul Richard Conteh, Enrico Boero MD, Paola Crida, Paolo Narcisi MD

Emergency Telephone Number: **+39 3472809804**

**1. INTRODUCTION**

We are inviting you to participate in this research study because you have been sent to Radiology department for an ultrasound (US) exam at Holy Spirit Hospital - Makeni. We’re collecting some information about your diagnosis and your treatment with and without the help of US. This information could include, eventually some US images, but would never include anything that would allow you to be identified by name.

# 2. YOUR PARTICIPATION IS VOLUNTARY

Your participation is entirely voluntary, so it is up to you to decide whether or not to take part in this study. Before you decide, it is important for you to understand what the research involves. This consent form will tell you about the study, why the research is being done, what will happen to you during the study and the possible benefits, risks and discomforts.

If you wish to participate, you will be asked to sign this form. If you do decide to take part in this study, you are still free to withdraw at any time and without giving any reasons for your decision.

If you do not wish to participate, you do not have to provide any reason for your decision not to participate nor will you lose the benefit of any medical care to which you are entitled or are presently receiving.

Please take time to read the following information carefully and to discuss it with your family, friends, and doctor before you decide.

**3.** **WHO IS CONDUCTING THE STUDY?**

Principal Investigator, Dr. Alessandro Lamorte, is conducting the study. The Principal Investigator and the co-investigators does not have any actual or potential conflicts of interest and will not receive financial compensation for enrolling subjects into this study.

**4. BACKGROUND**

Lacking of diagnostic devices is one of the main health problems in Sierra Leone. Train new ultrasonographers with the point-of-care technique could improve the diagnostic potential of every hospital in which there is an ultrasound machine.

# 5. WHAT IS THE PURPOSE OF THE STUDY?

The purpose of this study is to determine whether the integration of a new diagnostic instrument, in a low resources environment, can improve the diagnostic ability of the physicians that have in charge the patient. It will be studied as well the impact of ultrasonography on the number of referral, the kind of therapy and the x-ray imaging prescribed.

# 6. WHO CAN PARTICIPATE IN THE STUDY?

Participants should be sent to Ultrasound and be age more then 18 years.

7. WHO SHOULD NOT PARTICIPATE IN THE STUDY?

Everybody can voluntarily participate in this study

# 8. WHAT DOES THE STUDY INVOLVE?

This study will take place at Holy Spirit Hospital and will involve at least 50 volunteer subjects. You will receive routine treatment for your disease not different from that of standard treatment. The physician will fill a pre-ultrasound survey form, then you will be scanned and, in the end, the physician will fill a post- ultrasound survey form. During your exam some extra information collected about you in the course of normal care provided may also be used for a screening project about arterial hypertension. This information could include your blood pressure and your heart rate but would never include anything that would allow you to be identified by name. If you decide not to take part in the study your care will not be affected.

### **9. WHAT ARE MY RESPONSIBILITIES?**

This is an observational study and does not require your direct participation, other than the release of your information for research purposes.

# 10. WHAT ARE THE POSSIBLE HARMS AND SIDE EFFECTS OF PARTICIPATING?

Since this is an observational study involving an exam that would be done in any case, there are no risks associated with this study. The information the participants reveal will not affect their health care of the support given to them.

**11. WHAT ARE THE BENEFITS OF PARTICIPATING IN THIS STUDY?**

No one knows whether or not you will benefit from this study. There may or may not be direct benefits to you from taking part in this study. We hope that the information learned from this study can be used in the future to benefit other people and the Sierra Leone health system.

**12. WHAT ARE THE ALTERNATIVES TO THE STUDY TREATMENT?**

All subjects will receive the same diagnostic treatment as they would receive should they not participate in the study.

**13. WHAT HAPPENS IF I DECIDE TO WITHDRAW MY CONSENT TO PARTICIPATE?**

Your participation in this research is entirely voluntary. You may withdraw from this study at any time. If you decide to enter the study and to withdraw at any time in the future, there will be no penalty or loss of benefits to which you are otherwise entitled, and your future medical care will not be affected.

The study doctors/investigators may decide to discontinue the study at any time, or withdraw you from the study at any time, if they feel that it is in your best interests.

If you choose to enter the study and then decide to withdraw at a later time, all data collected about you during your enrolment in the study will be retained for analysis. By law, this data cannot be destroyed.

14. WHAT HAPPENS IF SOMETHING GOES WRONG?

Signing this consent form in no way limits your legal rights against the investigators, or anyone else.

15. REMUNERATION/COMPENSATION

Subjects will be not paid for their participation in this study.

**16. WILL MY TAKING PART IN THIS STUDY BE KEPT CONFIDENTIAL?**

Your confidentiality will be respected. No information that discloses your identity will be released or published without your specific consent to the disclosure. However, research records and medical records identifying you may be inspected in the presence of the Investigator or his or her designate by representatives for the purpose of monitoring the research. However, no records which identify you by name or initials will be allowed to leave the Investigators' locked offices.

**17.** **WHO DO I CONTACT IF I HAVE QUESTIONS ABOUT THE STUDY DURING MY PARTICIPATION?**

If you have any questions or desire further information about this study before or during participation, you can contact Dr. Alessandro Lamorte at +393472809804 or alessandro.lamorte@unito.it

# Sierra Leone Ultrasound Rainbow4Africa Project (SLURP)

#### **CONSENT STATEMENT**

- My signature on this form signifies that I have read and understood this subject information and consent form and that I freely give my consent to participate in this study.
- I have had sufficient time to consider the information provided and to ask for advice if necessary.
- I have had the opportunity to ask questions and have received satisfactory responses to my questions.
- I understand that all of the information collected will be kept confidential and that the results will only be used for scientific objectives.
- I understand that my participation in this study is voluntary and that I am completely free to refuse to participate or to withdraw at any time without changing in any way the quality of care that I receive.
- I understand that I am not waiving any of my legal rights as a result of signing this consent form.
- I understand that there is no guarantee that this study will provide any benefits to me (if applicable).
- I have read this form and I freely consent to participate in this study.
- I have been told that I will receive a dated and signed copy of this form.

_________________________ ____________________________ _______________

Name (printed) Signature Date

_________________________ ____________________________ _______________

Principal Investigator/designate Signature Date

(printed)
