# Supplementary material for: The Sierra Leone Ultrasound Rainbow4Africa Project (SLURP): an observational study of ultrasound effectiveness in developing countries
Source: Crit Ultrasound J. 2016 Oct 26;8:14. doi: 10.1186/s13089-016-0051-y (PMC5080275; doi:10.1186/s13089-016-0051-y)
Supplement: Supplementary file 2 — Additional file 2. HSH ultrasound data sheet. [file 13089_2016_51_MOESM2_ESM.pdf]

HSH Ultrasound Data Sheet pre-US

Clinician should fulfill only the grey-shadowed boxes and write in capital letters

1 Patient

Surname		Date of birth	__/__/____
Name		Date of enrollment	__/__/____
Sex	☐ M ☐ F	Serial number	
Vitals	BP: _____/_____ mmHg HR: ________ bpm SpO_2: ________ %		

2 Clinician requesting US scan

Name	Dr.
Main complaint of the patient (sign one)	☐ Abdominal pain<br>☐ Abdominal distension and discomfort<br>☐ Cough<br>☐ Palpitation<br>☐ Swelling of feet<br>☐ Chest pain and burns<br>☐ Dyspnea<br>☐ Menstrual problems<br>☐ Obstetrics problems<br>☐ Difficulty in passing urine<br>☐ Other (specify ____)
What is you **pre US differential diagnosis** list? (sign 1 or more)	☐ 1. PUD<br>☐ 2. Pneumonia<br>☐ 3. Pelvic muscle syndrome<br>☐ 4. Pleural Effusion<br>☐ 5. Cardiomiophaty<br>☐ 6. Liver cirrosis/Schistosomiasis<br>☐ 7. Cholecystolithiasis<br>☐ 8. Gastritis/GERD<br>☐ 9. Acute/Chronic nephritis<br>☐ 10. Nephrolithiasis/Idronephrosis<br>☐ 11. Uterus Myomatosis<br>☐ 12. Adnexitis / GUT Infection<br>☐ 13.Other (specify ______________________<br>____________________________________)

How **certain** is the ordering physician about the patient's diagnosis **prior** to the ultrasound scan?

☐ 1.Very uncertain ☐ 2. Uncertain ☐ 3.Exactly midway ☐ 4.Somewhat certain ☐ 5.Very certain

3 Therapy

Proposed **without** US	☐ Medical therapy ☐ Surgical therapy ☐ Referral to other Hospital
Therapy would consist in: (short description)	

4 US scan

Type of study requested (sign 1 or more)	☐ Cardiac superficial<br>☐ Chest<br>☐ Abdomen<br>☐ Ob/Gyn<br>☐ Soft tissue<br>☐ Other (specify ____________________)

HSH Ultrasound Data Sheet post-US

Clinician should fulfill only the grey-shadowed boxes and write in capital letters

5

**Patient name**	

6 — **Clinician after US scan**

Name	Dr.	
What is you **post US differential diagnosis** list? (sign 1 or more)	☐ 1. PUD<br>☐ 2. Pneumonia<br>☐ 3. Pelvic muscle syndrome<br>☐ 4. Pleural Effusion<br>☐ 5. Cardiomiophaty<br>☐ 6. Liver cirrosis/Schistosomiasis<br>☐ 7. Cholecystolithiasis	☐ 8. Gastritis/GERD<br>☐ 9. Acute/Chronic nephritis<br>☐ 10. Nephrolithiasis/Idronephrosis<br>☐ 11. Uterus Myomatosis<br>☐ 12. Adnexitis / GUT Infection<br>☐ 13.Other (specify ________________________<br>____)
Did US scan add **unexpected** diagnosis to this patient?	☐ Yes (specifiy ____________________________________) ☐ No	

How **certain** is the ordering physician about the patient's diagnosis **after** to the ultrasound scan?				
☐ 1.Very uncertain	☐ 2. Uncertain	☐ 3.Exactly midway	☐ 4.Somewhat certain	☐ 5.Very certain

7 — **Therapy**

Proposed **with** US	☐ Medical therapy	☐ Surgical therapy	☐ Referral to other Hospital
Therapy would consist in: (short description)			

8 — **Comments**

Hypothetically, how did this study affect your management of this patient? (free description	
